# How are we evaluating the cost-effectiveness of companion biomarkers for targeted cancer therapies? A systematic review

**DOI:** 10.1186/s12885-021-08725-4

**Published:** 2021-09-01

**Authors:** Mikyung Kelly Seo, John Cairns

**Affiliations:** 1 Department of Health Services Research and Policy, Faculty of Public Health and Policy, London School of Hygiene and Tropical Medicine, London, UK; 2 Centre for Cancer Biomarkers (CCBIO), University of Bergen, Bergen, Norway; 3 Department of Surgery and Cancer, Faculty of Medicine, Imperial College London, London, UK; 4 Department of Public Health and Primary Care, School of Clinical Medicine, University of Cambridge, Cambridge, UK

**Keywords:** Biomarker, Companion biomarker, Companion diagnostic, Precision medicine, Targeted therapy, Oncology, Cancer, Economic evaluation, Health economics, Cost-effectiveness, Systematic review

## Abstract

**Background:**

Despite the increasing economic assessment of biomarker-guided therapies, no clear agreement exists whether existing methods are sufficient or whether different methods might produce different cost-effectiveness results. This study aims to examine current practices of modeling companion biomarkers when assessing the cost-effectiveness of targeted cancer therapies. It investigates the current methods in modeling the characteristics of companion diagnostics based on existing economic evaluations of biomarker-guided therapies in cancer.

**Methods:**

A literature search was performed using Medline, Embase, EconLit, Cochrane library for economic evaluations of biomarker-guided therapies with companion diagnostics in cancer. Preferred Reporting Items of Systematic Reviews and Meta-Analyses (PRISMA) guidelines were followed. Studies were selected using pre-specified eligibility criteria based on the PICO framework. To make the included studies more comparable, we qualitatively synthesized the data under nine domains of methods where consensus was deemed lacking.

**Results:**

Only four of the twenty-two studies included in this review were found to be of good quality with respect to incorporating the characteristics of companion biomarkers in economic evaluations. However, many evaluations focused on a pre-selected patient group rather than including all patients regardless of their biomarker status. Companion biomarker characteristics captured in evaluations were often limited to the cost or the accuracy of the test. Often, only the costs of biomarker testing were modelled. Clinical outcomes and health state utilities were often not included due to the limited data generated by clinical trials. Methods of economic evaluation were not applied consistently in assessments of companion cancer biomarkers for targeted therapies. It was also shown that conflicting cost-effectiveness results were likely depending on what comparator arm was chosen and what comparison structure was designed in the model.

**Conclusion:**

We found no consistent approach applied in assessing the value of companion biomarker tests and including the characteristics of biomarkers in an economic evaluation of targeted oncology therapies. Currently, many economic evaluations fail to capture the full value of companion biomarkers beyond sensitivity/specificity and cost related to biomarker testing.

**Supplementary Information:**

The online version contains supplementary material available at 10.1186/s12885-021-08725-4.

## Introduction

Economic evaluations (EEs) are increasingly used to inform decisions regarding market access, reimbursement and coverage of new medical technologies including biomarker diagnostics for targeted therapies. Companion biomarkers are used to select and guide the best treatment options for patients prior to administering a corresponding therapy. However, no agreement exists whether existing methods are sufficient to evaluate the health economic impact of biomarkers, or whether different methodological approaches might produce conflicting results concerning the cost-effectiveness of biomarker-guided therapies.

This study focuses on companion biomarker tests for targeted cancer therapies (i.e. companion diagnostics for guided therapies in cancer). Specific biomarkers, known as companion diagnostics (CDx) are the focus of this review. CDx can be defined as a medical device (often in vitro) providing information regarding the safe and effective use of a corresponding intervention [1]. CDx is the diagnostic test labelled to be used prior to the administration of a particular therapeutic product and thus, the treatment decision is made based on the biomarker testing result. That is, the use of a specific test is obligatorily proceeded by the provision of corresponding therapy (e.g. HER2 testing prior to trastuzumab). If test accuracy is not satisfactory, the treatment decision can be detrimental to the patient outcomes when treated with the biomarker-guided therapy.

Given the indirect impact of companion diagnostics on the cost-effectiveness of biomarker-guided therapies, the EEs of test-guided therapies need to incorporate not only the characteristics of a medicine but also those of a test. In other words, for a companion test to achieve the improvement of patient health outcomes, the test must result in a change in the administration of its subsequent therapy. By influencing on the choice of a subsequent therapy, the companion test can indirectly improve health outcomes by delivering the right treatment to the right patient. It can then lead to the improved treatment effect of the corresponding guided therapy. Therefore, the EEs of the biomarker-guided therapy should capture the full spectrum of the co-dependency of the medicine that interacts with the companion biomarker test that assists in determining the right patient groups. However, there seems to be no agreement existent in the EE approaches for this type of co-dependent technologies. Consequently, few countries provided health EE methods guide specific to the co-dependent technologies such as companion diagnostics for biomarker-guided therapies [2, 3].

This study aims to investigate current practices of modeling and incorporating the characteristics of companion biomarker tests when assessing the cost-effectiveness of biomarker-guided therapies. It analyses the approaches currently adopted in EEs and highlights the current challenges and issues to be overcome to reach a consensus on methods and data requirements for EEs of companion diagnostics for biomarker-guided therapies.

## Methods

A systematic review of health economic evaluations of companion diagnostics for targeted cancer therapies was undertaken. This review was conducted following the recommendations of the Preferred Reporting Items of Systematic Reviews and Meta-Analyses (PRISMA) guidelines [4, 5].

### Literature search

A systematic literature search for EEs of cancer biomarkers co-licensed to administer targeted therapies (hereafter, called “companion biomarkers”) was conducted using Medline (Ovid), Embase (Ovid), EconLit, Cochrane library. A hand search of article citations and review articles identified a further four articles [6–9].

The electronic search was performed using Medical subject heading (MeSH) terms and keywords that were developed for disease (cancer), intervention (companion biomarkers for targeted therapies), and study design (economic evaluations). These were combined with free-word text searches using relevant economic terms (e.g. “cost-effectiveness”) and the names of biomarker-guided therapeutic products both in brand and generic terms. The CDx approved by the US.

Food and Drug Administration (FDA) [10] were targeted in the literature search. Studies published in English were searched from 2014 to February 2021. The 7-year search period was chosen given that this literature review aimed to explore current EE practice and to critically appraise them in depth. Seven years were considered to be long enough to capture a sufficient number of recently published EEs and also to exclude any approaches not applicable to current practice. Search terms are provided (Additional file 1).

### Study selection

Studies were selected using prespecified inclusion and exclusion criteria (Table  1) based on the PICOS (Population, Intervention, Comparator, Outcome, Study design) framework. Given the aims of this literature review, studies failing to report important information relevant to EEs of a companion biomarker test (e.g. biomarker characteristics, biomarker-related modeling inputs) were excluded.

**TABLE 1:** PICOS and Inclusion/Exclusion Criteria

PICOS	Inclusion criteria	Exclusion criteria
**Population**	Patients with cancer treated with biomarker-guided therapies.	Patients with cancer not treated with biomarker-guided therapies.
**Intervention**	Companion diagnostics. Biomarker-guided therapies. Precision medicine. Personalized medicine.	No companion diagnostics No predictive biomarkers for targeted therapies. No biomarker-guided therapies. Drugs without assessing companion biomarkers. Universal screening tools. Triage procedures. Severity or progression analyses.
**Comparator**	Unrestricted.	No comparative treatment. Surgery.
**Outcome**	Biomarker characteristics. Methodological approaches. Modeling inputs.	–
**Study type**	Full-text economic evaluations (cost-effectiveness analysis, cost-utility analysis).	Abstract. No economic evaluations. No English. Costing studies. Cost-minimization studies.

election of papers followed the eligibility criteria below:

**Population**: Patients with cancer tested with companion biomarker diagnostics for targeted therapies. Studies conducted on pre-specified patients with a particular biomarker status were excluded if they did not consider any of CDx-related characteristics in their evaluations

**Intervention**: companion biomarkers for targeted anti-cancer therapies. These biomarkers are used as diagnostic tools to guide the optimal treatment option(s) for patients responsive or unresponsive to the corresponding therapeutic products. Biomarker tests without market authorizations co-licensed with companion therapeutic products were not of interest in this review

**Comparator**: conventional treatments (e.g. chemotherapy, best supportive care) or targeted therapies with or without the use of companion biomarker tests

**Outcome**: Methodological or modelling approaches, biomarker characteristics, data inputs of biomarker tests. Studies without sufficient information reported on these items (e.g. abstracts) were excluded

**Study type**: economic evaluations including model or trial-based analyses

The study selection had three stages. First, the articles identified from the electronic databases were imported into EndNote® and duplicate citations removed. Second, the title and abstracts of the identified articles were screened to assess suitability by the first reviewer (MKS) and the studies clearly indicated as irrelevant were excluded. However,any studies with ambiguity were discussed with the second reviewer (JC). Third, the remaining articles that met the inclusion criteria were read in full text by the first reviewer (MKS) and cross-checked by the second reviewer (JC). Disagreements at any stage were resolved by discussion between the two reviewers (MKS, JC).

### Data analysis and synthesis

This review of current practice with respect to the EE of companion biomarkers focuses on nine methodological areas. These key areas were formulated based on previous studies and existing HTA documentation guides on co-dependent technologies [2, 3, 11–15], the Consolidated Health Economic Evaluation Reporting Standards (CHEERS) checklist [16]. We first used the framework of the CHEERS checklist and it provided useful information in formulating the key method areas for this review such as target population, study perspective, comparators, preference-based outcomes, and estimating resource use and costs. However, the CHEERS checklist alone was not sufficient to encompass the full spectrum of the characteristics of companion diagnostics for biomarker-guided therapies. Therefore, other information found in existing studies [13, 14, 17] and governmental documents [2, 3] was adopted in order to reflect the indirect impact of companion biomarker tests on patient health outcomes. For example, evidence on the measurement of the differential impact of the diagnostic on patient health outcomes needed to be considered. Thus, the EEs should incorporate the evidence of the test’s performance (or diagnostic accuracy) that result in a change in the management of subsequent therapeutic service. Also, it was observed from our previous empirical studies that the structure of comparing alternative strategies and choosing comparator strategy in EEs might lead to different cost-effectiveness results of their corresponding test-guided therapies and health outcomes [14, 15, 18].

The nine domains framed for the synthesis of this review are following: (i) target population; (ii) study perspective; (iii) structure of comparing alternative strategies; (vi) measurement of clinical value of companion biomarkers; (v) measurement and valuation of preference-based outcomes of companion biomarker tests; (vi) estimating resource use and costs; (vii) timing of the test use; (viii) uncertainty analysis; (ix) data sources for biomarker-related data inputs. The narrative syntheses and analyses were performed.

for these ninemethodological areas. To be more specific, a list of questions was developed based on these items (Additional file 2). 

## Results

We initially identified 2544 potential studies. After removing duplicates and reviewing titles and abstracts, 100 publications were included for full-text screening. 78 papers were found to be not eligible for inclusion according to pre-defiined inclusion/exclusion criteria (Table 1). A considerable number of publications (*n* = 21) had to be excluded because they did not consider any characteristics of companion biomarker tests in their EEs of test-guided therapies. Finally, twenty-two papers found to be relevant and included in this review. Details are provided in PRISMA diagram (Additional file 3).

**Fig. 1:**
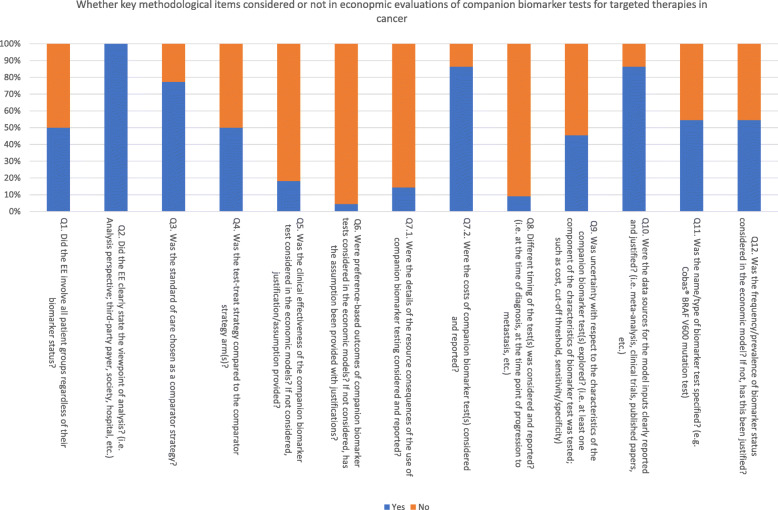
Graph of including the characteristics of companion biomarkers in economic evaluation

Characteristics of the included studies are detailed in Table  2. Figure 1 provides the synthesized overview of whether the key methodological areas were addressed or not in the evaluations. The model inputs that were most frequently missing, related to companion biomarker tests, were preference-based outcomes, clinical utility, resource use, and the timing of the test. A detailed analysis of the key methodological areas by publication is provided in additional file 4.

**Table 2:** Detailed characteristics of the included studies

Study	Objective	Biomarker test	Corresponding therapy compared	Strategies compared	Biomarker related model inputs considered	Country	Perspecti-ve	Model type	Time horizon	Outcome measure	Funding
Aguiar 2017 [19]	To assess cost-effectiveness of immune checkpoint inhibitor with and without the use of PD-L1 testing for patient selection.	PD-L1 expression.	Immunotherapy (Nivolumab, Pembrolizumab, Atezolizumab)	3 strategies compared: Treat-all with docetaxel. Treat-all with immunotherapy. Test-treat (if PD-L1 expressed with 1% or more, patients were treated with immunotherapy; if not, treated with docetaxel.)	PD-L1 testing cost. PD-L1 expression cut-off points (PD-L1 > 1% used in base-case analysis, while 5, 10and 50% tested in sensitivity analysis.)	USA	Third-party payer (Medicare) .	Decision- analytic model. (No further details given.)	5-year horizon	QALY	No funding declared.
Bhadhuri 2019 [20]	To evaluate the cost-effectiveness of pembrolizumab monotherapy compared with chemotherapy for metastatic NSCLC with PD-L1 tumour progression score (TPS) > = 50%.	PD-L1 expression.	Pembrolizumab.	2 strategies compared on pre-specified patients expressing high levels of PD-L1: Treat-all with pembrolizumab. Treat-all with chemotherapy. However, a secondary analysis was also performed using a ‘test-treat’ strategy where patients tested for PD-L1 expression and treated with pembrolizumab if the test determined PD-L1 TPS > = 50%.	PD-L1 testing cost.	Switerzland	Third-party payer.	Partitioned survival model.	20-year horizon. (tested for 5, 10, 30 years).	QALY	Commerical funding.
Chouaid 2017 [21]	To assess the cost-effectiveness of afatinib versus gefitinib for EGFR mutation-positive NSCLCs.	EGFR mutation.	Afatinib, Gefitinib.	2 strategies compared on pre-specified patients: Treated with afatinib. Treated with gefitinib.	EGFR testing cost.	France	Third-party payer.	Partitioned survival model.	10-year horizon.	QALY	Commercial funding.
Curl 2014 [22]	To compare three strategies (dacarbazine, vemurafenib, vemurafenib plus ipilimumab) for patients with BRAF positive metastatic melanoma.	BRAF mutation.	Dacarbazine, Vemurafenib, Vemurafenib plus Ipilimumab	3 strategies compared on pre-specified patients: Treated with dacarbazine. Treated with vemurafenib. Treated with vemurafenib plus ipilimumab.	BRAF testing cost (Cobas®)	USA	Third-party payer (Medicare).	Decision tree model.	Lifetime	QALY	No funding.
Dottino 2019 [23]	To determine the cost-effectiveness of a poly (ADP-ribose) polymerase (PARP) inhibitor for the maintenance treatment of recurrent ovarian cancer.	BRCA mutation.	Niraparib.	4 maintenance strategies compared: Observation (no treatment). Test-treat with gBRAC testing. Test-treat with BRAC plus HRD (homologuous recombination deficiency) testing. Treat-all with PARP inhibitor/niraparib.	gBRCA testing cost. Prevalence of gBRAC mutations.	USA.	Third-party payer (Medicare)	Decision analysis model.	24 months (trial period).	Progression-free QALY	Grant funding.
Ewara 2014 [24]	To assess the cost-effectiveness of three strategies (bevacizumab plus FOLFIRI, cetuximab plus FOLFIRI, panitumumab plus FOLFIRI) for mCRC patients with KRAS WT.	KRAS mutation.	Bevacizumab, Cetuximab, Panitumumab.	3 strategies compared on pre-specified patients: Treated with bevacizumab plus FOLFIRI. Treated with cetuximab plus FOLFIRI. Treated with panitumumab plus FOLFIRI.	KRAS testing cost.	Canada	Third-party payer.	Markov model	100-month horizon.	QALY	No funding.
Genuino 2019 [25]	To assess the cost-effectiveness and budget impact of adjuvant trastuzumab therapy for HER2-positive early-stage breast cancer.	HER2 expression.	Trastuzumab.	2 strategies were compared on pre-specified patients with HER2-positive: Treat all with trastuzumab and chemotherapy. Treat all with chemotherapy only.	Percentage of HER2-positivity of breast cancer in the Philippines.	Philippines.	HCS and societal perspective.	Markov model.	Lifetime.	QALY	No funding.
Graham 2014 [26]	To assess the cost-effectiveness of panitumumab plus mFOLFOX6 compared with bevacizumab plus mFOLFOX6.	RAS mutation.	Panitumumab, Bevacizumab.	2 strategies compared on pre-specified patients: Treated with panitumumab plus mFOLFOX6. Treated with bevacizumab pus mFOLFOX6.	KRAS and RAS testing cost. RAS frequency.	USA	Third-party payer.	Semi-Markov model.	Lifetime	QALY	Commercial funding.
Graham 2016 [27]	To assess the cost-effectiveness of subsequent-line treatment with cetuximab or panitumumab in patients with WT KRAS mCRC.	KRAS mutation.	Cetuximab, Panitumumab.	2 strategies compared on pre-specified patients: Treated with cetuximab. Treated with panitumumab.	KRAS testing cost.	USA	Third-party payer.	Semi-Markov model.	Lifetime	QALY	Commercial funding.
Harty 2018 [28]	To investigate the clinical effectiveness and cost-effectiveness of panitumumab plus chemotherapy and cetuximab plus chemotherapy for rat scarcoma (RAS) wild-type (WT) patients for the first-line treatment of mCRC.	KRAS/RAS mutation.	Cetuximab.	2 strategies compared on patients expressing EGFR: Treated with FOLFIRI alone. Treated with cetuximab plus FOLFIRI.	EGFR testing cost. RAS testing cost. Sensitivity/specificity not considered but all patients were presumed to be correctly stratified because these biomarker testing techniques have high technical accuracy.	UK	Third-party payer.	Markov model.	10-year horizon.	QALY	Commercial funding.
Holleman 2020 [29]	To compare the cost-effectiveness of first line gefitinib, erlotinib, afatinib, and osimertinib in patients with EGFR-mutated NSCLC.	EGFR mutation.	Gefitinib, Erlotinib, Afatinib, Osimertinib.	4 strategies were compared on pre-specified patients with EGFR-positive: Treat all with gefitinib. Treat all with erlotinib. Treat all with afatinib. Treat all with osimertinib.	EGFR mutation testing cost. Frequency of EGFR mutations in Dutch patients with NSCLC (11%) was mentioned but unclear if it was incorporated in the model.	Netherlands	Societal perspective.	Markov model.	Lifetime.	QALY.	No funding.
Huxley 2017 [30, 31]	To investigate the clinical effectiveness and cost-effectiveness of panitumumab plus chemotherapy and cetuximab plus chemotherapy for rat scarcoma (RAS) wild-type (WT) patients for the first-line treatment of mCRC.	RAS mutation.	Cetuximab, Panitumumab.	5 strategies compared on pre-specified patients: Treated with FOLFOX/FOLFIRI. Treated with cetuximab plus FOLFOX/FOLFIRI. Treated with panitumumab plus FOLFOX.	RAS testing cost. RAS prevalence (50% of patients assumed to be RAS wild-type).	UK	Third-party payer.	Markov model.	30-year horizon.	QALY	Governmental funding.
Janmaat 2016 [32]	To determine the ICER of adding cetuximab to first-line chemotherapeutic treatment of patients with advanced esophageal squamous cell carcinoma (ESCC), based on RCT II trial.	EGFR expression.	Cetuximab.	2 strategies compared on pre-specified patients expressing EGFR: Treated with cetuximab plus cisplatin-5-fluorouracil. Treated with cisplatin-5-fluorouracil.	EGFR testing cost. EGFR prevalence (60% patients assumed to be EGFR positive).	Netherlands	Third-party payer.	Monte Carlo simulation using individual patient data.	0.9 years.	QALY	No funding.
Lim 2016 [33]	To evaluate the cost-effectiveness of treating patients guided by EGFR testing compared to no-testing (which is current practice in South Korea).	EGFR expression.	Erlotinib.	2 strategies compared: Test-treat (if EGFR positive, treated with erlotinib; if EGFR wild-type, treated with conventional chemotherapy; if unknown, re-biopsy required). No-testing (Treat all with conventional chemotherapy).	EGFR testing cost (Therascreen®, Cobas®). Testing accuracy (sensitivity/specificity).	South Korea.	Third-party payer.	Markov model.	5-year horizon.	QALY	Governmental funding.
Lu 2016 [8]	To examine the economic outcome of three techniques for testing ALK gene rearrangement combining with crizotinib (first-line), compared with traditional regimen.	ALK gene rearrangement.	Crizotinib.	3 ALK rearrangement testing techniques prior to crizotinib were compared (4 strategies compared): No gene screening - all treated with standard chemotherapy. Ventana IHC - if ALK rearrangement positive, treated with crizotinib; if ALK rearrangement negative, treated with standard chemotherapy. qRT-PCR - if ALK rearrangement positive, treated with crizotinib; if ALK rearrangement negative, treated with standard chemotherapy Conventional IHC - if IHC ALK rearrangement negative, treated with standard chemotherapy; if IHC ALK rearrangement positive, FISH testing (to confirm) to be performed and then, if FISH ALK rearrangement negative, treated with standard chemotherapy, if FISH ALK rearrangement positive, treated with crizotinib.	Cost of ALK rearrangement testing (Ventana IHC; IHC; qRT-PCR; FISH) Sensitivity and specificity respectively for Ventana IHC; IHC; qRT-PCR). ALK prevalence	China	Third-party payer.	Markov model.	10-year horizon.	QALY	Commercial funding.
Lu 2018 [34]	To evaluate the cost-effectiveness of ALK tests (two genes-guided testing) followed by crizotinib for advanced NSCLC compared to standard chemotherapy.	ALK rearrangement.	Crizotinib.	3 strategies compared: No gene screening - all treated with standard chemotherapy. NGS panel tests - if ALK rearrangement positive, treated with crizotinib; if ALK rearrangement negative, treated with standard chemotherapy. Multiplex PCR testing - if ALK positive, treated with crizotinib; if negative, treated with standard chemotherapy.	ALK prevalence. Cost of ALK rearrangement testing (NGS, Multiplex PCR). Sensitivity and specificity (NGS panel tests assumed to be 100%; multiplex PCR testing obtained from a published literature).	China	Third- party payer.	Markov model.	10-year horizon.	QALY	Commercial funding.
Morgan 2017	To assess the cost-effectiveness of crizotinib in untreated anaplastic lymphoma kinase-positive (ALK-positive) non-small-cell-lung cancer (NSCLC).	ALK expression.	Crizotinib.	2 strategies compared on pre-specified patients with ALK-positive NSCLC: Treat all with crizotinib. Treat all with pemetrexed chemotherapy in combination with cisplatin or carboplatin.	ALK testing cost ImmunoHistoChemistry (IHC) testing cost Fluorescence in situ hybridisation (FISH) testing cost	UK	Third-party payer.	‘area-under-the curve’ Markov model.	15-year horizon	QALY	Governmental funding.
Saito 2017	To determine the cost-effectiveness of comprehensive molecular profiling before initiating anti-EGFR therapies in mCRC.	RAS mutation. Comprehensive profiling that includes PTEN + ERBB2, PTEN + SRC, and BRAF + RNF43 mutations (CancerPlex®).	Bevacizumab, Panitumumab.	3 strategies compared: No testing RAS screening Comprehensive screening	Biomarker testing cost. Proportion of molecular subgroups (proportion of patients per biomarker status).	Japan	Third-party payer.	Markov model	5-year horizon.	QALY	Unclear (Not reported)
Wen 2015	To explore the costs and effectiveness of RAS screening before monoclonal antibodies in mCRC based on FIRE-3 study.	RAS mutation.	Cetuximab, Bevacizumab.	Four strategies compared on pre-specified patients (FIRE3 trial patients with KRAS wild type status): KRAS tested - treated with cetuximab and FOLFIRI. RAS tested - treated with cetuximab and FOLFIRI. KRAS tested - treated with bevacizumab and FOLFIRI. RAS tested - treated with bevacizumab and FOLFIRI.	KRAS/RAS testing cost.	China	Third-party payer.	Markov model.	10-year horizon.	QALY	No funding.
Westwood 2014	To compare the performance and cost-effectiveness of KRAS mutation tests in differentiating adults with mCRC who may benefit from first-line treatment of cetuximab in combination with standard chemotherapy from those who should receive standard chemotherapy alone.	KRAS mutation.	Cetuximab.	10 different tests for KRAS mutation status. No comparator approach taken. Cobas KRAS Mutation Test Kit (Roche Molecular Systems). Therascreen KRAS RGQ PCR Kit (QIAGEN). Therascreen KRAS Pyro Kit (QIAGEN). KRAS LightMix Kit (TIB MOLBIOL). KRAS StripAssay (ViennaLab). HRM analysis. Pyrosequencing. MALDI-TOF mass spectrometry. Next-generation sequencing. Sanger sequencing.	KRAS testing cost. KRAS testing accuracy (sensitivity/specificity) KRAS prevalence (KRAS mutant, KRAS wild-type, KRAS unknown test result). Timing of the test – justifications given.	UK	Third-party payer.	Markov model	Lifetime (23 years)	QALY	Governmental funding.
Wu 2017	To evaluate the economic outcome of adding cetuximab to the standard chemotherapy.	RAS mutation.	Cetuximab.	2 strategies compared: No testing – treat all with FLOFIRI. Test-treat (if RAS wild-type, treated with cetuximab plus FOLFIRI, if RAS mutant, treated with FOLFIRI).	RAS testing cost. RAS prevalence.	China	Third-party payer.	Markov model.	Lifetime	QALY	No funding.
Zhou 2016	To evaluate the cost-effectiveness of predictive testing for extended RAS WT status in the context of targeting the use of cetuximab/bevacizumab.	RAS mutation.	Cetuximab, Bevacizumab.	4 strategies compared on pre-specified patients (CALGB 80405 trial patients with KRAS wild type status): KRAS WT tested-treated with cetuximab plus chemotherapy. KRAS WT tested-treated with bevacizumab plus chemotherapy. RAS WT tested-treated with cetuximab plus chemotherapy. RAS WT tested-treated with bevacizumab plus chemotherapy.	KRAS/RAS testing cost.	China	Societal perspective.	Markov model.	Lifetime	QALY	No funding.

The most frequently used modeling type was a Markov model (thirteen papers), followed by partitioned survival model (three papers) and semi-Markov model (two papers). All economic evaluations were performed from a third-party payer perspective except for two studies which took a societal perspective and one study done on both perspectives. All studies were performed for high-income countries except for five studies of China and one of Philippines.

### Target population

The patient population targeted in EEs of biomarker-guided therapies was varied, but fall into two broad categories; patients with a known biomarker status, and patients whose biomarker status is initially unknown. Fourteen studies were performed on a pre-defined group of patients with particular biomarker status [20–22, 24–30, 32, 35–37]; however, they considered at least one characteristic of companion biomarker tests in their evaluations. Many EEs were conducted using a pre-specified patient group with particular confirmed biomarker status, and authors used this to justify excluding some of the key characteristics of companion biomarker testing from their evaluations.

### Study perspective

The study perspective defines the scope of costs and health benefits to be assessed in an EE. All included studies clearly reported their perspective. A majority of studies showed that EEs were performed applying the third-party payer perspective. Only three studies stated that they employed a societal perspective [25, 29, 37]; two from low-and-middle income countries and one from a high income country. Meanwhile, two USA studies [22, 23] were found to be more appropriately described as a third-party payer perspective (i.e. Medicare) although authors stated that their studies were analysed from the societal perspective.

Given the nature of multiple purposes of biomarker testing application or use, and the indirect impact of companion biomarker diagnostics on patient health benefits, a third party payer perspective might not be sufficient to capture all costs and benefits relevant to companion biomarkers when identifying patients suitable for the corresponding therapy. However, only two studies considered indirect costs such as travel fees and absenteeism costs, together with the cost of adverse events [29, 37]. However, this study did not consider any biomarker-related indirect costs either. For example, Schnell-Inderst and colleagues conducted a targeted review and highlighted measuring the potential effect modifiers such as the dependency of treatment effects on contextual factors and learning curve [38].

### The strategies compared

It is widely accepted that current practice with respect to the target population is a relevant alteranative strategy with which to compare [39, 40].

**Fig. 2:**
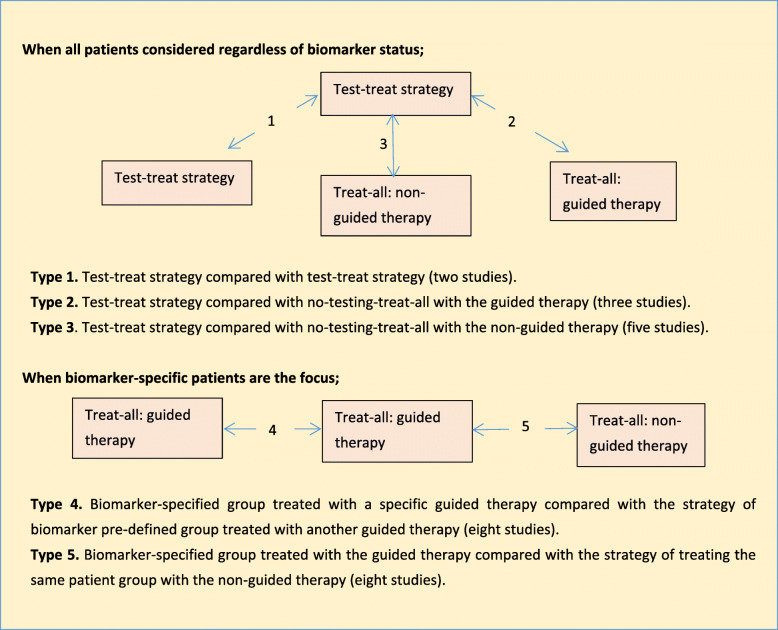
Strategies in EEs of companion biomarker therapies

Five different types of comparison have been undertaken in the literature evaluating the use of companion biomarkers in order to guide treatment in cancer. Three of these occur when all patients considered regardless of their biomarker status, and two types of comparison have been made when the focus is on patients with a specific biomarker status (Fig. 2).

A total of eight studies featured involved test-treat strategies, where those testing positive would receive the guided therapy and those testing negative would receive the non-guided therapy. Two studies [6, 41] compared different test-treat strategies (Type 1). Three studies [6, 20, 23] compared a test-treat strategy with no testing where all patients were treated with the guided therapy (Type 2). Five studies [8, 23, 33, 34, 42] compared a test-treat strategy with no testing where all patients received the non-guided therapy (Type 3).

Fifteen studies considered patients with a specific biomarker status. Eight studies [21, 24, 26, 27, 29, 30, 36, 37] involved the comparison of two (or more) different guided therapies (Type 4). Eight studies [19, 20, 22, 25, 28, 30, 32, 35] compared patients receiving a guided therapy with treatment with a non-guided therapy (Type 5). Except for Huxley et al. [30], all of these studies only considered one characteristic of the companion biomarker test (usually the cost of testing). The details are prevented in Fig. 2 and Additional file 5.

### Measuring the clinical value of companion biomarkers

No consensus currently exists on data requirements when incorporating the clinical value of biomarkers into the modeling of EEs of biomarker-guided therapies. For example, the Diagnostic Assessment Programme requires consideration of the diagnostic accuracy in the appraisal of diagnostic tests [43], although it is not always feasible in practice especially when assessors are not presented with any data on test accuracy. On the other hand, the NICE methods guide for technology appraisal does not necessarily require test accuracy but requires inclusion of the associated costs of biomarker testing [39]. Furthermore, none of the EEs reviewed examined the accuracy of a companion biomarker diagnostic test separately, for example by testing different cut-off thresholds including false positive and false negative results as part of uncertainty analysis. The cut-off threshold is the cut-off point defining the presence of the biomarker, determining biomarker-positive and biomarker-negative patients for the administration of corresponding co-dependent therapeutic agents [44–46]. Varying levels of accuracy may lead to different patient subgroups being eligible for the corresponding drugs. According to previous studies [13, 47], the clinical value of biomarker tests could be assessed in three ways; analytic validity, clinical validity, and clinical utility. Analytic validity concerns how well a test detects the presence or absence of a particular marker [40]. Clinical validity refers to the performance of a test (diagnostic accuracy) in detecting the presence of a specific disorder; so-called sensitivity and specificity [13]. Clinical utility is defined in the ACCE (analytical validity, clinical validity, clinical utility, and ethical/legal/social implications) model project as “how likely the test is to significantly improve patient outcomes”, which goes beyond sensitivity and specificity and then which may change treatment options for the patient [48]. In other words, clinical utility (effectiveness) of companion testing technology is based on the ability to improve patient health outcomes by altering treatment decisions [49, 50].

Relatively few EEs considered the diagnostic accuracy of biomarker testing using data on sensitivity and specificity [8, 33, 34, 41]. Many EEs did not consider the performance of biomarker testing or often did not mention this at all [6, 19–27, 29, 32, 37]. Otherwise, some studies provided some assumptions or justifications why they did not consider the clinical value of a companion diagnostic test [28, 30, 35, 36, 42]. It is often assumed that the technical accuracy of patient stratification by biomarker testing is perfect and thus, the sensitivity and specificity were either not considered or assumed to be 100%. However, no studies explicitly considered or assumed the clinical utility of companion biomarkers in their EEs. For example, no studies stated that the clinical value of companion biomarker testing was supposedly incorporated into the clinical effectiveness of the corresponding drug based on the clinical trial of the sub-population delineated by the diagnostic.

Meanwhile, a handful of studies considered the frequency or prevalence of a particular biomarker status among their target patient populations [6, 8, 23, 25, 26, 30, 32, 34, 41, 42]. Among them, only one study considered the probability of an unknown test result in the analysis [41].

### Measurement and valuation of preference-based outcomes

The quality-adjusted life-year (QALY) is a preference-based health outcome widely used in EEs of therapeutic products [51, 52]. It is widely accepted because it allows comparisons of health benefits and costs across different disease areas and therapeutic interventions. However, challenges emerge with the economic assessment of companion biomarkers given the nature of targeted therapies guided by companion biomarker testing and indirect impact of companion biomarker testing on patient outcomes. The current metrics for measuring preference-based outcomes using population-based preferences cannot fully capture patient preferences for biomarker tests [53]. There seem to be more aspects of individual patient preference when valuing biomarker tests for guided therapies rather than conventional non-guided drugs. For example, patients could be informed in advance of the likelihood of therapeutic response or unresponsiveness prior to the provision of treatment.

Patients can have an improved sense of controlling their own choices of therapeutic options informed by their biomarker status rather than left with uncertainty on whether to have the treatment or not. Shared decision making (SDM) and communication between patients and clinicians will put patients at the centre of treatment decisions guided by companion biomarker test results. Patients may feel empowered to make informed decisions about their own treatment and care [54–56]. Although the provision of biomarker-guided therapy is dictated by the patient’s biomarker status, being informed of the biomarker status can support the SDM of both clinicians and patients to explore more fully the potential benefits and risks. It can then potentially improve patient satisfaction with health services.

Companion diagnostics for cancer patients usually require collecting a bio-sample for analysis, with potential implications for process utility (including reassurance or information) [57–59]. Brennan and Dixon [60] report different approaches being used to detect and measure process utility such as gamble techniques, time trade-off, and conjoint analysis. Some biomarker tests involve relatively invasive methods to collect the bio-sample, such as tissue biopsy, needle biopsy, skin biopsy in diagnosing cancer [61, 62], that can be measured and incorporated into QALY estimates. Yet, how to measure and incorporate process utility into cost-utility analyses needs to be further researched with more empirical studies in HTA. If companion biomarker tests were already integrated into the clinical study of measuring patient-reported outcomes (PROs) for co-dependent therapeutic agents, it can be assumed that the disutility or utility value of companion biomarker testing is already embedded or indirectly expressed in PROs of the corresponding therapy. Yet, this aspect should be transparently reported in health economic models of companion biomarkers or biomarker-guided therapies. Nevertheless, none of the EEs included in this systematic review discussed these aspects of companion biomarker testing or indicated how preference-based outcomes of companion biomarker devices were measured and valued. For example, no studies explicitly included utility or disutility values for biomarker testing. Where biomarker testing uses tissues collected in a previous biopsy, it can be argued that patient preferences do not need to be considered in economic modeling. However, none of the EEs mentioned this aspect or attempted to justify the omission of preference-based outcomes of biomarker testing. As an example, patients might need to undergo another biopsy for the purpose of biomarker testing after cancer has progressed to metastasis, or a second biopsy might be needed to confirm the biomarker status when the testing accuracy was unsatisfactory,or the turnaround time for the biomarker testing may lead to additional waiting time for patients to access the treatment,or patients might experience anxiety or hopelessness when informed that the test predicts non-response to the targeted therapy and no alternative therapy options are available.

### Estimating resource use and costs

All included EE studies considered the costs of biomarker testing; however, some details were absent. Some papers did not report the cost of biomarker testing devices [19, 20, 25] and often a lump sum cost was modelled without providing details on how the total cost calculated [21, 22, 24, 32, 36, 37]. Several studies reported at least some details regarding the data source or the names/types of biomarker testing kits [6, 7, 23, 26–30, 33–35, 42], but many EEs did not consider or report the resource use parameters relevant to the testing of companion biomarkers. None of the studies considered the capital cost related to the initial purchase of a biomarker test kit or diagnostic equipment as well as other costs such as training staff, relevant consumables, or lab reporting tools. Even in the situation where laboratories can re-purpose existing testing platforms to deliver the new test, relevant costs of consumables and staff with appropriate skills need to be considered. As an example, the NICE committee was aware that ALK testing would be not carried out in this specific clinical setting if crizotinib was not available [63], and therefore it is highly likely that hospitals would need to purchase testing equipment, however, this was not considered in the EE.

### Timing of the test use

Details of where in the clinical pathway testing was undertaken were often not reported. Only two studies [6, 41] provided some explanation on this aspect; however, it was not clear how the timing of the test use was considered in the analysis of the Westwood study [64]. Whereas Saito and colleagues [6] provided and justified their assumptions. Given the nature of companion biomarkers, the patient’s health benefit arises from the corresponding therapy guided by the testing result, which is best understood as part of the clinical pathway in relation to its indirect impact on patient outcomes. Therefore, companion biomarkers’ value is best assessed while considering the timing of the test use; for example, whether the testing was done at diagnosis or following progression to metastasis. Westwood and colleagues [41] noted that KRAS testing’s timing might vary; some clinicians might undertake routine testing for all patients at diagnosis or some might wait until metastases have been detected. Yet, they did not specify how their evaluation was done in this respect.

### Uncertainty analysis

Six studies [19, 28–30, 32–34] explored the impact of cost-effectiveness of varying at least one component of the characteristics of companion biomarker tests being evaluated such as unit cost, total testing cost, test accuracy, cut-off thresholds, and biomarker prevalence. However, many studies did not examine a test’s characteristics separately from that of the corresponding therapy. According to one HTA guideline, “if a diagnostic test to establish the presence or absence of the biomarker is carried out solely to support the treatment decision … a sensitivity analysis should be provided without the cost of the diagnostic test” [39]. However, out of four UK studies, two studies performed a sensitivity analysis on biomarker testing cost [28, 30].

### Data sources for biomarker-related data inputs

All but three studies [19, 24, 32] provided data sources used for biomarker tests’ characteristics. However, several studies did not identify a specific companion biomarker testing kit, although some of them reported a general biomarker testing type (e.g. RAS testing) and therefore, several studies were not transparent and reproducible. The most frequently used data sources were previously published literature. However, testing cost inputs were mostly sourced from reimbursement schedules [22, 23, 27, 28, 32, 42], manufactures or laboratories [26, 37, 41], and if such information was unavailable, expert opinions were sought [30, 35].

## Discussion

Altogether, twenty-two papers were included in this review. One systematic review similar in terms of study scope and objectivemainly focused on reviewing the sensitivity and specificity of companion diagnostics and the testing costs [12]. It did not provide a comprehensive review of methodological approaches to EEs for assessing the value for money of companion biomarkers in the context of precision medicine. To the best of our knowledge, this is the first review providing a comprehensive report on current practices and possible solutions in terms of methodological approaches and evidence requirements in assessing the value for money of companion biomarkers. Table 3 summaries possible solutions and suggestions for the methodological issues identified in this review.

**TABLE 3:** Summary of current practices and solutions in economic evaluations of companion biomarkers

Methodological areas	Issues identified in the current practice of economic evaluations	Possible solutions/suggestions	
		Methodological approaches	Data requirements
**Target population**	Pre-selected population group with known biomarker status was targeted in EEs.	Target the entire patient group including biomarker positive, negative, and unknown.	Clinical data on all patients including false positive, false negative, unknown biomarker status.
**Perspective**	Payer perspective was mostly used following the HTA guidelines by the reimbursement authority.	Holistic viewpoint desired (e.g. societal perspective). However, if infeasible, biomarker testing related cost items should be included in evaluations.	Cost data collected from administrative database or real-world setting.
**Comparator**	With versus without the use of biomarker testing compared in evaluations yet in the context of the same targeted therapy.	SOC in current routine clinical practice should be employed as a comparator in the context of treating the disease condition of interest and the target patient population.	Evidence on standard of care being routinely practiced for the target patient population with the disease condition in a country-specific setting.
**Comparison structure**	No consistency in structuring strategies to be compared in comparative analysis of companion biomarkers for targeted therapies.	Test-treat versus treat-all with SOC is suggested as a base-case comparison structure.	Clinical data on patients treated all with SOC without biomarker tested. Clinical data on patients tested negative.
**Clinical effectiveness**	Clinical value of companion biomarkers was limited to sensitivity/specificity. Often, biomarker prevalence data was ignored. Sensitivity /specificity was often assumed to be 100% or excluded completely from the economic model inputs.	Clinical value of companion biomarkers beyond sensitivity /specificity should be incorporated in economic evaluations of biomarker-guided therapies.	Clinical evidence generated from clinical trials on both the drug and the diagnostic. If possible, separate RCTs in test positive and test negative patients respectively treated with guided therapy and non-guided therapy. In addition, the clinical utility values including the change of clinician’s behavior in choosing this treatment option over SOC should be captured.
**Preference-based outcome**	Utility and/or disutility values related to biomarker testing were not considered.	Biomarker related patient preferences should be incorporated in economic evaluations of biomarker-guided therapies.	Individual patient utility (or disutility) values on the use of a companion biomarker test prior to the administration of targeted therapy. Patient preference data can be acquired along the clinical trials, reflecting all biomarker relevant preference items.
**Timing of the test use**	The timing of the use of companion biomarker testing is often not incorporated and not reported in economic evaluations.	The value of companion biomarkers should be understood throughout the clinical pathways applicable to the decision-making of clinicians.	The timing of the test use in clinical routine settings is preferred over the RCT setting.
**Uncertainty analysis**	Many economic evaluations did not examine the characteristics of a test separately from that of the corresponding therapy.	The characteristic components relevant to a companion biomarker diagnostic should be tested separately as part of uncertainty analysis of biomarker-guided therapy.	Value of information analysis can be useful to inform the uncertainty around current information/data against perfect or partial perfect information.
**Information and model inputs to be incorporated in economic evaluations of companion biomarkers**	Limited number of model parameters pertinent to biomarker testing was incorporated into the economic assessment of companion biomarkers.	Model inputs relevant to companion biomarker testing should all be captured and incorporated in economic evaluations of biomarker-guided therapies.	Name/type of biomarker testing diagnostic/kit. Resource use of testing. Unit cost of testing. Capital cost if the testing device is not currently available in current clinical settings. Prevalence of biomarker status in patient population. Sensitivity/specificity. Utility and/or disutility values of performing the test in relation to preference-based outcomes. Clinical pathways including the test (for example, when the test is performed in routine clinical settings).

Many of the EEs of biomarker-guided therapies focus on a pre-selected patient group instead of including all patients with a disease regardless of their biomarker status. This is then often used as a justification for excluding companion biomarker testing from EE, leading to a lack of robust economic evidence for the entire patient group with the disease. It is important to consider all patients regardless of biomarker status and perform the economic assessment of companion biomarker therapies for all populations of interest with the condition or disease.

Also, EEs need to be consistent with the decision problem being addressed for targeted patient populations using a payer perspective. EEs usually adopt a perspective proposed in country-specific health technology assessment guidelines and then, the third-party payer perspective is the most frequently employed viewpoint of analysis. However, considering the multiple purposes of biomarker tests and the indirect health impact of companion biomarkers on patient outcomes of corresponding therapies, it might be better to adopt a holistic viewpoint and capture the full spectrum of biomarkers’ health economic consequences. This would then permit the inclusion of non-health-related costs and benefits such as early information or reassurance on a treatment option.

Applying the comparator strategy of relevance in specific clinical settings is crucial and may change the cost-effectiveness outcomes of the intervention being assessed. The economic evaluation of biomarker-guided therapies often requires more than one comparator arm such as biomarker-guided therapy without biomarker testing and standard of care without biomarker testing [17]. A previous study [14] sometimes found conflicting cost-effectiveness results depending on the comparator strategy chosen such as test-treat versus treat-all with the standard of care (SOC) and test-treat versus treat-all with the new therapy. We found no consistency in the choice of comparator strategies and in structuring the strategies to be compared. Biomarker-guided therapies were often evaluated by comparing biomarker testing and no-testing strategies to administer the new intervention. Such comparative analyses often ignore the standard of care being provided in current clinical practice.

These issues appear to be linked to one another. As found in this study, many EEs of biomarker-guided therapies do not necessarily consider the entire patient population; it instead narrows down to a specific patient group with known biomarker status. And this narrowed-down population leads to a narrower scope of the decision problem being addressed by EEs, which may not be necessarily congruent with the interest of decision-makers (i.e. payers) for their reimbursement decision-making of the entire patient group. Furthermore, this narrowed-down scope of a decision problem and a patient population group appears to be used to justify the inconsistent approaches in structuring the alternative strategies and incorporating the characteristics of companion tests in their EEs of biomarker-guided therapies. For example, a considerable number of studies focused on the population of biomarker-specified patients and justified their comparative structure of ‘treat-all with guided therapy’ versus ‘treat-all with non-guided therapy’ while incorporating only very limited data inputs related to the companion diagnostics such as testing cost only. Likewise, although the test’s performance (i.e. diagnostic accuracy) is a key element of modeling companion diagnostics in EEs, the information for patients with false negative and false positive results was often ignored or blindly justified by the narrowed-scope of patient populations with known biomarker status. These lead to further ignorance of key characteristics of companion biomarker tests such as key epidemiological data like the biomarker prevalence or mutation in the population level.

Meanwhile, generating the evidence for improved health outcomes is not always straightforward. If companion biomarker tests are integrated into the clincal trials of their guided therapies, then it can assume that their clinical utility is already reflected in the clinical evidence for the corresponding guided therapies [65]. Otherwise, it is not easy to show the clinical utility of companion biomarkers in clinical practice. In other words, the clinical utility of companion biomarker tests is indirectly expressed in the patient outcome of their co-dependent therapies. However, often, biomarker tests are developed independently from the drug, and the common practice of biomarker test developers in terms of evidence generation is only limited to provide clinical validity (i.e. sensitivity and specificity). Reflecting this common practice in the generation of clinical evidence for biomarkers, we found that assessing the clinical value of companion biomarkers in EEs is limited to a consideration of the sensitivity and specificity of the test.

Most studies considered and included the cost of companion biomarker testing in their EEs. However, they often did not provide sufficient details on how they calculated the cost of testing and what data sources were used. This posed challenges in terms of transparency and reproducibility of EEs of companion biomarkers. This may be because the testing cost is not standardized (e.g. no coding systems exist for biomarker testing in medical records) or not publicly available (e.g. secret pricing or individually negotiated price at a hospital/laboratory level) in many countries. Given that no standardized cost information such as unit costs is publicly available, most economic evaluations might need to rely on laboratory charges.

It is said, in the field of precision medicine, that we need to introduce more flexible reimbursement systems to reward innovation, reflecting the added value of diagnostics or biomarker tests [66]. Otherwise, the value of biomarkers will not be fully captured and reflected in EEs. This also leads to an issue of understanding the entire clinical pathway in relation to the biomarker test and capturing the added value of biomarkers along the continuum of disease management and cure. Our study showed that many evaluations failed to reflect this aspect by not even reporting the timing of the test. Furthermore, the impact of companion biomarker tests in terms of HRQoL or adverse events was largely ignored.

## Conclusion

It is in the public interest to ensure timely integration of new technologies into clinical use through adequate reimbursement and coverage levels. However, this requires that test developers demonstrate robust evidence of the health economic impact of biomarker tests. Companion biomarker characteristics captured in EEs are often limited to the cost or the accuracy of the test. Often, only the costs of biomarker testing are modelled. Clinical outcomes or utilities are often difficult to include due to the limited data generated by clinical trials.

We found that there was no consistent approach applied in assessing the value of biomarkers and including the characteristics of biomarkers in an economic evaluation of targeted oncology therapies. Currently, many EEs fail to capture the full range of characteristics that influence the value of companion biomarkers beyond testing cost and sensitivity/specificity.

## Supplementary Information

**Additional file 1.** Search strategy/search terms.

**Additional file 2.** List of key methodological items in reviewing the EEs of biomarker-guided therapies.

**Additional file 3.** PRISMA flow diagram of study selection.

**Additional file 4.** List of including the characteristics of companion biomarkers in the economic evaluations.

**Additional file 5.** Summary of comparative analysis structure employed in EEs of companion biomarker therapies.

## Data Availability

All data generated or analysed during this study are included in this published article and its additional files.

## Abbreviations

CDx: Companion Diagnostics
EE: Economic Evaluation
FDA: Food and Drug Administration
PICOS: Population, Intervention, Comparator, Outcome, Study design
PRISMA: Preferred Reporting Items of Systematic Reviews and Meta-Analyses
CHEERS: Consolidated Health Economic Evaluation Reporting Standards

## References

[1] US Food and Drug Administration. Companion Diagnostic. https://www.fda.gov/medical-devices/vitro-diagnostics/companion-diagnostics Accessed 12 May 2019.

[2] Australian Government Deparment of Health. Technical guidelines for preparing assessment reports for the medical services advisory committee - service type: Investigative (Version 3.0). Available at http://www.msac.gov.au/internet/msac/publishing.nsf/Content/0BD63667C984FEEACA25801000123AD8/$File/InvestigativeTechnicalGuidelines-December-2016-Version-3.0.pdf [Accessed 14 November 2019]. In: Health Do, editor 2017.

[3] Scottish Medicine Consortium (SMC). New product assessment form. Available at https://www.scottishmedicines.org.uk/making-a-submission/ [Accesssed 25 April 2020]. 2019.

[4] Liberati A, Altman DG, Tetzlaff J, Mulrow C, Gøtzsche PC, Ioannidis JP (2009). The PRISMA statement for reporting systematic reviews and meta-analyses of studies that evaluate health care interventions: explanation and elaboration. J Clin Epidemiol.

[5] Moher D, Liberati A, Tetzlaff J, Altman DG (2010). Preferred reporting items for systematic reviews and meta-analyses: the PRISMA statement. Int J Surg.

[6] Saito S, Kameyama H, Muneoka Y, Okuda S, Wakai T, Akazawa K (2017). Cost-effectiveness analysis of the use of comprehensive molecular profiling before initiating monoclonal antibody therapy against metastatic colorectal cancer in Japan. J Cancer Policy.

[7] Butzke B, Oduncu FS, Severin F, Pfeufer A, Heinemann V, Giessen-Jung C, Stollenwerk B, Rogowski WH (2016). The cost-effectiveness of UGT1A1 genotyping before colorectal cancer treatment with irinotecan from the perspective of the German statutory health insurance. Acta Oncol.

[8] Lu S, Zhang J, Ye M, Wang B, Wu B (2016). Economic analysis of ALK testing and crizotinib therapy for advanced non-small-cell lung cancer. Pharmacogenomics..

[9] Squires H, Stevenson M, Simpson E, Harvey R, Stevens J (2016). Trastuzumab Emtansine for treating HER2-positive, Unresectable, locally advanced or metastatic breast Cancer after treatment with Trastuzumab and a Taxane: an evidence review group perspective of a NICE single technology appraisal. Pharmacoeconomics..

[10] US Food and Drug Administration. List of cleared or approved companion diagnostic devices (In vitro and imaging tools). Available from: https://www.fda.gov/medical-devices/vitro-diagnostics/list-cleared-or-approved-companion-diagnostic-devices-vitro-and-imaging-tools [Last accessed 27 November 2018].

[11] Blanchard A, Strand R (2017). Cancer biomarkers: ethics, economics and society.

[12] Doble B, Tan M, Harris A, Lorgelly P (2015). Modeling companion diagnostics in economic evaluations of targeted oncology therapies: systematic review and methodological checklist. Expert Rev Mol Diagn.

[13] Annemans L, Redekop K, Payne K (2013). Current methodological issues in the economic assessment of personalized medicine. Value Health.

[14] Seo MK, Cairns J (2018). Do cancer biomarkers make targeted therapies cost-effective? A systematic review in metastatic colorectal cancer. PLoS One.

[15] Seo MK, Straume O, Akslen LA, Cairns J (2020). HSP27 expression as a novel predictive biomarker for bevacizumab: is it cost effective?. PharmacoEconomics - Open.

[16] Husereau D, Drummond M, Petrou S, Carswell C, Moher D, Greenberg D, Augustovski F, Briggs AH, Mauskopf J, Loder E, ISPOR Health Economic Evaluation Publication Guidelines-CHEERS Good Reporting Practices Task Force (2013). Consolidated health economic evaluation reporting standards (CHEERS)—explanation and elaboration: a report of the ISPOR health economic evaluation publication guidelines good reporting practices task force. Value Health.

[17] Faulkner E, Annemans L, Garrison L, Helfand M, Holtorf A-P, Hornberger J, Hughes D, Li T, Malone D, Payne K, Siebert U, Towse A, Veenstra D, Watkins J, Personalized Medicine Development and Reimbursement Working Group (2012). Challenges in the development and reimbursement of personalized medicine—payer and manufacturer perspectives and implications for health economics and outcomes research: a report of the ISPOR personalized medicine special interest group. Value Health.

[18] D’Avó Luís AB, Seo MK. Has the development of cancer biomarkers to guide treatment improved health outcomes? The European Journal of Health Economics. 2021. 10.1007/s10198-021-01290-4 PMC8214594 33783662

[19] Aguiar PN, Perry LA, Penny-Dimri J, Babiker H, Tadokoro H, de Mello RA (2017). The effect of PD-L1 testing on the cost-effectiveness and economic impact of immune checkpoint inhibitors for the second-line treatment of NSCLC. Ann Oncol.

[20] Bhadhuri A, Insinga R, Guggisberg P, Panje C, Schwenkglenks M (2019). Cost effectiveness of pembrolizumab vs chemotherapy as first-line treatment for metastatic NSCLC that expresses high levels of PD-L1 in Switzerland. Swiss Med Wkly.

[21] Chouaid C, Luciani L, LeLay K, Do P, Bennouna J, Perol M, Moro-Sibilot D, Vergnenègre A, de Pouvourville G (2017). Cost-effectiveness analysis of Afatinib versus Gefitinib for first-line treatment of advanced EGFR-mutated advanced non-small cell lung cancers. J Thorac Oncol.

[22] Curl P, Vujic I, van’t Veer LJ, Ortiz-Urda S, Kahn JG. Cost-effectiveness of treatment strategies for BRAF-mutated metastatic melanoma. PLoS One. 2014;9 (9) (no pagination)(e107255). 10.1371/journal.pone.0107255 PMC4157865 25198196

[23] Dottino JA, Moss HA, Lu KH, Secord AA, Havrilesky LJ (2019). U.S. Food and Drug Administration-approved poly (ADP-ribose) polymerase inhibitor maintenance therapy for recurrent ovarian Cancer: a cost-effectiveness analysis. Obstet Gynecol.

[24] Ewara EM, Zaric GS, Welch S, Sarma S (2014). Cost-effectiveness of first-line treatments for patients with KRAS wild-type metastatic colorectal cancer. Curr Oncol.

[25] Genuino AJ, Chaikledkaew U, Guerrero AM, Reungwetwattana T, Thakkinstian A (2019). Cost-utility analysis of adjuvant trastuzumab therapy for HER2-positive early-stage breast cancer in the Philippines. BMC Health Serv Res.

[26] Graham CN, Hechmati G, Hjelmgren J, De Liege F, Lanier J, Knox H (2014). Cost-effectiveness analysis of panitumumab plus mFOLFOX6 compared with bevacizumab plus mFOLFOX6 for first-line treatment of patients with wild-type RAS metastatic colorectal cancer. Eur J Cancer.

[27] Graham CN, Maglinte GA, Schwartzberg LS, Price TJ, Knox HN, Hechmati G, Hjelmgren J, Barber B, Fakih MG (2016). Economic analysis of Panitumumab compared with Cetuximab in patients with wild-type KRAS metastatic colorectal Cancer that progressed after standard chemotherapy. Clin Ther.

[28] Harty G, Jarrett J, Jofre-Bonet M. Consequences of biomarker analysis on the cost-effectiveness of Cetuximab in combination with FOLFIRI as a first-line treatment of metastatic colorectal Cancer: personalised medicine at work. Applied health economics and health policy. 2018:1–11. 10.1007/s40258-018-0395-5 PMC6028886 29948926

[29] Holleman MS, Al MJ, Zaim R, Groen HJM, Uyl-de Groot CA (2020). Cost-effectiveness analysis of the first-line EGFR-TKIs in patients with non-small cell lung Cancer Harbouring EGFR mutations. Eur J Health Econ.

[30] Huxley N, Crathorne L, Varley-Campbell J, Tikhonova I, Snowsill T, Briscoe S (2017). The clinical effectiveness and cost-effectiveness of cetuximab (review of technology appraisal no. 176) and panitumumab (partial review of technology appraisal no. 240) for previously untreated metastatic colorectal cancer: A systematic review and economic evaluation. Health Technol Assess.

[31] Tikhonova IA, Huxley N, Snowsill T, Crathorne L, Varley-Campbell J, Napier M, Hoyle M (2018). Economic analysis of first-line treatment with Cetuximab or Panitumumab for RAS wild-type metastatic colorectal Cancer in England. Pharmacoeconomics..

[32] Janmaat VT, Bruno MJ, Polinder S, Lorenzen S, Lordick F, Peppelenbosch MP, et al. Cost-effectiveness of cetuximab for advanced esophageal squamous cell carcinoma. PLoS One. 2016;11 (4) (no pagination)(e0153943). 10.1371/journal.pone.0153943 PMC4839693 27100871

[33] Lim EA, Lee H, Bae E, Lim J, Shin YK, Choi SE. Economic evaluation of companion diagnostic testing for EGFR mutations and first-line targeted therapy in advanced non-small cell lung cancer patients in South Korea. PLoS One. 2016;11 (8) (no pagination)(e0160155). 10.1371/journal.pone.0160155 PMC4970739 27483001

[34] Lu S, Yu Y, Fu S, Ren H. Cost-effectiveness of ALK testing and first-line crizotinib therapy for non-small-cell lung cancer in China. PLoS One. 2018;13 (10) (no pagination)(e0205827). 10.1371/journal.pone.0205827 PMC6198972 30352060

[35] Morgan P, Woolacott N, Biswas M, Mebrahtu T, Harden M, Hodgson R. Crizotinib for untreated anaplastic lymphoma kinase-positive non-small-cell lung Cancer: an evidence review group perspective of a NICE single technology appraisal. Pharmacoeconomics. 2017:1–11. 10.1007/s40273-017-0497-1 28342113

[36] Wen F, Yang Y, Zhang P, Zhang J, Zhou J, Tang R, Chen H, Zheng H, Fu P, Li Q (2015). Cost-effectiveness of RAS screening before monoclonal antibodies therapy in metastatic colorectal cancer based on FIRE3 study. Cancer Biol Ther.

[37] Zhou J, Zhao R, Wen F, Zhang P, Tang R, Chen H, et al. Economic evaluation study (CHEER-compliant): Cost-effectiveness analysis of RAS screening for treatment of metastatic colorectal cancer based on the CALGB 80405 trial. Medicine (United States). 2016;95 (27) (no pagination)(e3762). 10.1097/MD.0000000000003762 PMC5058788 27399059

[38] Schnell-Inderst P, Hunger T, Conrads-Frank A, Arvandi M, Siebert U. Ten recommendations for assessing the comparative effectiveness of therapeutic medical devices: a targeted review and adaptation. J Clin Epidemiol. 2018;94:97–113.(doi):10.1016/j.jclinepi.2017.09.022. Epub Oct 28. 10.1016/j.jclinepi.2017.09.022 29107757

[39] National Institute of Health and Care Excellence. Guide to the methods of technology appraisal. 2013. Available: https://www.ncbi.nlm.nih.gov/books/NBK395867/pdf/Bookshelf_NBK395867.pdf [Last accessed: 10 May 2019]. 27905712

[40] Canadian Agency for Drugs and Technologies in Health. Guidelines for the economic evaluation of health technologies: Canada [3rd edition]. Ottawa: Canadian Agency for Drugs and Technologies in Health. Available http://www.inahta.org/wp-content/themes/inahta/img/AboutHTA_Guidelines_for_the_Economic_Evaluation_of_Health_Technologies.pdf. 2006.

[41] Westwood M, van Asselt T, Ramaekers B, Whiting P, Joore M, Armstrong N (2014). KRAS mutation testing of tumours in adults with metastatic colorectal cancer: A systematic review and cost-effectiveness analysis. Health Technol Assess.

[42] Wu B, Yao Y, Zhang K, Ma X (2017). RAS testing and cetuximab treatment for metastatic colorectal cancer: a cost-effectiveness analysis in a setting with limited health resources. Oncotarget..

[43] National Institute for Clinical Excellence (NICE). Diagnostic Assessment Programme Manual. 2011.: Available: https://www.nice.org.uk/Media/Default/About/what-we-do/NICE-guidance/NICE-diagnostics-guidance/Diagnostics-assessment-programme-manual.pdf [Last accessed: 23 April 2020].

[44] Fridlyand J, Simon RM, Walrath JC, Roach N, Buller R, Schenkein DP, Flaherty KT, Allen JD, Sigal EV, Scher HI (2013). Considerations for the successful co-development of targeted cancer therapies and companion diagnostics. Nat Rev Drug Discov.

[45] US Food and Drug Administration. Developing and labeling in vitro companion diagnostic devices for a specific group of oncology therapeutic products: Guidance for industry. Available at https://www.fda.gov/media/120340/download. 2020.

[46] Subtil F, Rabilloud M (2014). Estimating the optimal threshold for a diagnostic biomarker in case of complex biomarker distributions. BMC medical informatics and decision making.

[47] Seo MK. Economic evaluations of cancer biomarkers for targeted therapies: practices, challenges, and policy implications. In: Blanchard A, Strand R, editors. Cancer biomarkers: ethics, economics and society: Megaloceros Press; 2017. p. 25–38.

[48] National Academies of Sciences E, Medicine. Biomarker tests for molecularly targeted therapies: key to unlocking precision medicine: National Academies Press; 2016. 26962610

[49] Lesko L, Zineh I, Huang SM (2010). What is clinical utility and why should we care?. Clinical Pharmacology & Therapeutics.

[50] Sorich MJ, Coory M (2014). Interpreting the clinical utility of a pharmacogenomic marker based on observational association studies. The pharmacogenomics journal.

[51] Drummond MF, Sculpher MJ, Claxton K, Stoddart GL, Torrance GW (2015). Methods for the economic evaluation of health care programmes: Oxford university press.

[52] National Institute for Clinical Excellence (NICE). Guide to the methods of technology appraisal. https://www.niceorguk/process/pmg9/chapter/the-reference-case#exploring-uncertainty. 2013. 27905712

[53] Oosterhoff M, van der Maas ME, Steuten LM (2016). A systematic review of health economic evaluations of diagnostic biomarkers. Applied health economics and health policy.

[54] Coulter A, Collins A (2011). Making shared decision-making a reality.

[55] Charles C, Gafni A, Whelan T (1997). Shared decision-making in the medical encounter: what does it mean? (or it takes at least two to tango). Soc Sci Med.

[56] Weston WW (2001). Informed and shared decision-making: the crux of patient-centred care. Cmaj..

[57] Donaldson C, Shackley P (1997). Does “process utility” exist? A case study of willingness to pay for laparoscopic cholecystectomy. Soc Sci Med.

[58] Mooney G (1994). Key issues in health economics: harvester wheatsheaf.

[59] Gerard K, Mooney G (1993). QALY league tables: handle with care. Health Econ.

[60] Brennan VK, Dixon S (2013). Incorporating process utility into quality adjusted life years: a systematic review of empirical studies. Pharmacoeconomics..

[61] Mayo Clinic. Biopsy: Types of biopsy procedures used to diagnose cancer. https://www.mayoclinic.org/diseases-conditions/cancer/in-depth/biopsy/art-20043922 Accessed 7 June 2020 2020

[62] J Sorich M, A McKinnon R (2012). Personalized medicine: potential, barriers and contemporary issues. Curr Drug Metab.

[63] Centers for Disease Control Prevention. Genomic Testing: ACCE model process for evaluating genetic tests. CDCP, Atlanta (available online at: www. cdc. gov/genomics/gtesting/ACCE); 2010.

[64] Westwood M, Asselt A, Ramaekers B, Whiting P, Joore M, Armstrong N (2014). KRAS mutation testing of tumours in adults with metastatic colorectal cancer: a systematic review and cost-effectiveness analysis.

[65] National Institute of Health and Care Excellence. Crizotinib for previously treated anaplastic lymphoma kinase-positive advanced non-small-cell lung cancer. Technology Appraisal Guidance TA422. 21 December 2016. Available: https://www.nice.org.uk/guidance/ta422.

[66] Cohen JP, Felix AE. Personalized Medicine's bottleneck: diagnostic test evidence and reimbursement. J Pers Med. 2014;4(2):163–75. 10.3390/jpm4020163. 10.3390/jpm4020163 PMC4263971 25563222

