# Supplementary material for: How are we evaluating the cost-effectiveness of companion biomarkers for targeted cancer therapies? A systematic review
Source: BMC Cancer. 2021 Sep 1;21:980. doi: 10.1186/s12885-021-08725-4 (PMC8408935; doi:10.1186/s12885-021-08725-4)
Supplement: Supplementary file 1 — Additional file 1. Search strategy/search terms. [file 12885_2021_8725_MOESM1_ESM.docx]

**Additional file 1. Search strategy/search terms**

**Database: Medline**

1. Neoplasms/
2. Carcinomas/
3. Sarcomas/
4. Lymphoma/
5. Leukemia/
6. Germ cell tumors/
7. metastas$.mp. [mp=ti, ot, ab, nm, hw]
8. tumour$.mp. [mp=ti, ot, ab, nm, hw]
9. tumor$.mp. [mp=ti, ot, ab, nm, hw]
10. cancer$.mp. [mp=ti, ot, ab, nm, hw]
11. neoplasm$.mp. [mp=ti, ot, ab, nm, hw]
12. carcinoma$.mp. [mp=ti, ot, ab, nm, hw]
13. lymphoma$.mp. [mp=ti, ot, ab, nm, hw]
14. sarcoma$.mp. [mp=ti, ot, ab, nm, hw]
15. leukemia$.mp. [mp=ti, ot, ab, nm, hw]
16. (#1 OR #2 OR #3 OR #4 OR #5 OR #6 OR #7 OR #8 OR #9 OR #10 OR #11 OR #12 OR #13 OR #14 OR #15)
17. exp "Cost-Benefit Analysis"/
18. (value of life or economics, medical or economics, pharmaceutical or models, economic or markov chains or monte carlo method or uncertainty).sh.
19. economics.fs.
20. economics.sh.
21. ((econom$ or cost or costly or costing or costed or prices or pricing or discount or discounts or discounted or discounting or budget$ or afford$ or pharmacoeconomic$ or pharmaco) adj1 economic$).ti,ab.
22. (decision adj1 (tree$ or analy$ or model$)).ti,ab.
23. (#17 OR # 18 OR #19 OR #20 OR #21 OR #22)
24. (Lynparza* or olaparib). mp. [mp=ti, it, ab, nm, hw]
25. (Talzenna* or talazoparib). mp. [mp=ti, it, ab, nm, hw]
26. (Rubraca* or rucaparib). mp. [mp=ti, it, ab, nm, hw]
27. (Iressa* or gefitinib). mp. [mp=ti, it, ab, nm, hw]
28. (Gilotrif* or afatinib). mp. [mp=ti, it, ab, nm, hw]
29. (Vizimpro* or dacomitinib). mp. [mp=ti, it, ab, nm, hw]
30. (Tarceva* or erlotinib). mp. [mp=ti, it, ab, nm, hw]
31. (Tagrisso* or osimertinib). mp. [mp=ti, it, ab, nm, hw]
32. (Keytruda* or pembrolizimab). mp. [mp=ti, it, ab, nm, hw]
33. (Tecentriq* or atezolizumab). mp. [mp=ti, it, ab, nm, hw]
34. (Tibsovo* or ivosidenib). mp. [mp=ti, it, ab, nm, hw]
35. (Tasigna* or nilotinib). mp. [mp=ti, it, ab, nm, hw]
36. (Alecensa* or alectinib). mp. [mp=ti, it, ab, nm, hw]
37. (Xalkori* or crizotinib). mp. [mp=ti, it, ab, nm, hw]
38. (Zykadia* or ceritinib). mp. [mp=ti, it, ab, nm, hw]
39. (Tafinlar* or dabrafenib). mp. [mp=ti, it, ab, nm, hw]
40. (Mekinist* or trametinib). mp. [mp=ti, it, ab, nm, hw]
41. (Zelboraf* or vemurafenib). mp. [mp=ti, it, ab, nm, hw]
42. (Cotellic* or cobimetinib). mp. [mp=ti, it, ab, nm, hw]
43. (Herceptin* or trastuzumab). mp. [mp=ti, it, ab, nm, hw]
44. (Perjeta* or pertuzumab). mp. [mp=ti, it, ab, nm, hw]
45. (Kadcyla* or ado-trastuzumab emtansine)
46. (Erbitux* or cetuximab)
47. (Vectibix* or panitumumab)
48. (Idhifa* or enasidenib)
49. (Venclexta* or venetoclax)
50. (Gleevec* or Gilvec* or imatinib mesylate)
51. (Exjade* or deferasirox)
52. (#26 OR #27 OR #28 OR #29 OR #30 OR #31 OR #32 OR #33 OR #34 OR #35 OR #36 OR #37 OR #38 OR #39 OR #40 OR #41 OR #42 OR #43 OR #44 OR #45 OR #46 OR #47 OR #48 OR #49 OR #50 OR #51)
53. (#16 AND #23 AND #52)
54. Limits: humans, full-text, English, 7-year (2014-present)

**Database: Embase**

1. exp neoplasm
2. (neoplasm$) OR (metatas$) OR (carcinoma$) OR (sarcoma$) OR (lymphoma$) OR (leukemia) OR (germ adj3 cell adj3 tumor$) OR (tumo?r$) OR (cancer$)
3. (#1 OR #2)
4. exp "cost benefit analysis"
5. (Lynparza* or olaparib)
6. (Talzenna* or talazoparib)
7. (Rubraca* or rucaparib)
8. (Iressa* or gefitinib)
9. (Gilotrif* or afatinib)
10. (Vizimpro* or dacomitinib)
11. (Tarceva* or erlotinib)
12. (Tagrisso* or osimertinib)
13. (Keytruda* or pembrolizimab)
14. (Tecentriq* or atezolizumab)
15. (Tibsovo* or ivosidenib)
16. (Tasigna* or nilotinib)
17. (Alecensa* or alectinib)
18. (Xalkori* or crizotinib)
19. (Zykadia* or ceritinib)
20. (Tafinlar* or dabrafenib)
21. (Mekinist* or trametinib)
22. (Zelboraf* or vemurafenib)
23. (Cotellic* or cobimetinib)
24. (Herceptin* or trastuzumab)
25. (Perjeta* or pertuzumab)
26. (Kadcyla* or ado-trastuzumab emtansine)
27. (Erbitux* or cetuximab)
28. (Vectibix* or panitumumab)
29. (Idhifa* or enasidenib)
30. (Venclexta* or venetoclax)
31. (Gleevec* or Gilvec* or imatinib mesylate)
32. (Exjade* or deferasirox)
33. (#5 OR #6 OR #7 OR #8 OR #9 OR #10 OR #11 OR #12 OR #13 OR #14 OR #15 OR #16 OR #17 OR #18 OR #19 OR #20 OR #21 OR #22 OR #23 OR #24 OR #25 OR #26 OR #27 OR #28 OR #29 OR #30 OR #31 OR #32)
34. (#3 AND #4 AND #33)
35. Limits: humans, full-text, English, 7-year (2014-present)

**Database: Econlit**

1. biomark$ OR (molecu$ adj3 mark$) OR (tumo?r adj3 mark$) OR (biologic$ adj3 mark$) OR (signature adj3 molecule$)
2. (target adj3 therap$) OR (targeted adj3 therap$) OR (personali#ed adj3 medicine$) OR (companion adj3 diagnostic$ OR (precision adj3 medicine$) OR (codependent adj3 technolog$)
3. neoplasm$ OR metatas$ OR carcinoma$ OR sarcoma$ OR lymphoma$ OR leukemia OR (germ adj3 cell adj3 tumor$) OR tumo?r$ OR cancer$

Lynparza$ OR olaparib OR Talzenna$ OR talazoparib OR Rubraca$ OR rucaparib OR Iressa$ OR gefitinib OR Gilotrif$ OR afatinib OR Vizimpro$ OR dacomitinib OR Tarceva$ OR erlotinib OR Tagrisso$ OR osimertinib OR Keytruda$ OR pembrolizimab OR Tecentriq$ OR atezolizumab OR Tibsovo$ OR ivosidenib OR Tasigna$ OR nilotinib OR Alecensa$ OR alectinib OR Xalkori$ OR crizotinib OR Zykadia$ OR ceritinib OR Tafinlar$ OR dabrafenib OR Mekinist$ OR trametinib OR Zelboraf$ OR vemurafenib OR Cotellic$ OR cobimetinib OR Herceptin$ OR trastuzumab OR Perjeta$ OR pertuzumab OR Kadcyla$ OR ado-trastuzumab emtansine OR Erbitux$ OR cetuximab OR Vectibix$ OR panitumumab OR Idhifa$ OR enasidenib OR Venclexta$ or venetoclax OR Gleevec$ OR Gilvec$ OR imatinib mesylate OR Exjade$ OR deferasirox

1. (#1 or #2 or #3 or #4)
2. Limits: full-text, period (2014 to current)

**Database: Cochrane via Wiley (technology assessments and economic evaluations)**

1. MeSH descriptor: [Neoplasms] explode all trees
2. MeSH descriptor: [Sarcoma] explode all trees
3. MeSH descriptor: [Lymphoma] explode all trees
4. MeSH descriptor: [Leukemia] explode all trees
5. (neoplasm$) OR (metatas$) OR (carcinoma$) OR (sarcoma$) OR (lymphoma$) OR (leukemia) OR (germ adj3 cell adj3 tumor$) OR (tumo?r$) OR (cancer$)
6. (#1 OR #2 OR #3 OR #4 OR #5)
7. MeSH descriptor: [Costs and Cost Analysis] explode all trees
8. MeSH descriptor: [Cost-Benefit Analysis] explode all trees
9. MeSH descriptor: [Economics, Pharmaceutical] explode all trees
10. (cost effective$) OR (cost benefit$) OR (cost utility$) OR (economic evaluation$)
11. (#7 OR #8 OR #9 OR #10)
12. Lynparza$ or olaparib OR Talzenna$ or talazoparib OR Rubraca$ or rucaparib OR Iressa$ or gefitinib OR Gilotrif$ or afatinib OR Vizimpro$ or dacomitinib OR Tarceva$ or erlotinib OR Tagrisso$ or osimertinib OR Keytruda$ or pembrolizimab OR Tecentriq$ or atezolizumab OR Tibsovo$ or ivosidenib OR Tasigna$ or nilotinib OR Alecensa$ or alectinib OR Xalkori$ or crizotinib OR Zykadia$ or ceritinib OR Tafinlar$ or dabrafenib OR Mekinist$ or trametinib OR Zelboraf$ or vemurafenib OR Cotellic$ or cobimetinib OR Herceptin$ OR trastuzumab OR Perjeta$ or pertuzumab OR Kadcyla$ OR ado-trastuzumab emtansine OR Erbitux$ OR cetuximab OR Vectibix$ OR panitumumab OR Idhifa$ OR enasidenib OR Venclexta$ or venetoclax OR Gleevec$ or Gilvec$ or imatinib mesylate OR Exjade$ or deferasirox
13. (#6 AND #11 AND #12)
14. Limits: publication from Jan 2014 to present Cochrane protocols, Clinical answers, Editorials
