# Supplementary material for: How are we evaluating the cost-effectiveness of companion biomarkers for targeted cancer therapies? A systematic review
Source: BMC Cancer. 2021 Sep 1;21:980. doi: 10.1186/s12885-021-08725-4 (PMC8408935; doi:10.1186/s12885-021-08725-4)
Supplement: Supplementary file 2 — Additional file 2. List of key methodological items in reviewing the EEs of biomarker-guided therapies. [file 12885_2021_8725_MOESM2_ESM.docx]

**Additional file 2.** **List of key methodological items in reviewing the EEs of biomarker-guided therapies**

| **Question items** | **Yes** | **No** |
| --- | --- | --- |
| Q1. Did the EE include all patient groups regardless of their biomarker status (test positive, negative, unknown)? |  |  |
| Q2. Did the EE justify the viewpoint of analysis? (i.e. Analysis perspective; third-party payer, society, hospital, etc.) |  |  |
| Q3. Was the standard of care chosen as a comparator strategy? |  |  |
| Q4. Was the test-treat strategy compared to the comparator strategy arm(s)? |  |  |
| Q5. Was the clinical effectiveness of the companion biomarker test considered in the economic models? If not considered, justification/assumption provided? |  |  |
| Q6. Were preference-based outcomes of companion biomarker tests were considered in the economic models? If not considered, has the assumption been provided with justifications? |  |  |
| Q7-1. Were the details of the resource consequences of the use of companion biomarker testing considered and reported? |  |  |
| Q7-2. Were the costs of companion biomarker test(s) considered and reported? |  |  |
| Q8. Different timing of the test(s) was considered and reported? (i.e. at the time of diagnosis, at the time point of progression to metastasis, etc.) |  |  |
| Q9. Was uncertainty with respect to the characteristics of the companion biomarker test(s) explored? (i.e. at least one component of the characteristics of biomarker test was tested; such as cost, cut-off threshold, sensitivity/specificity) |  |  |
| Q10. Were the data sources for the model inputs clearly reported and justified? (i.e. meta-analysis, clinical trials, published papers, etc.) |  |  |
| Q11. Was the name/type of biomarker test specified? (e.g. Cobas® BRAF V600 mutation test) |  |  |
| Q12. Was the frequency/prevalence of biomarker status considered in the economic model? If not, has this been justified? |  |  |
