# Supplementary material for: How are we evaluating the cost-effectiveness of companion biomarkers for targeted cancer therapies? A systematic review
Source: BMC Cancer. 2021 Sep 1;21:980. doi: 10.1186/s12885-021-08725-4 (PMC8408935; doi:10.1186/s12885-021-08725-4)
Supplement: Supplementary file 3 — Additional file 3. PRISMA flow diagram of study selection. [file 12885_2021_8725_MOESM3_ESM.docx]

**Additional file 3. PRISMA flow diagram of study selection**

Screening

Included

Eligibility

Identification

Records identified through database searching (n = 2540)

Additional records identified through other sources (n = 4)

Duplicates removed (n = 119)

Records screened
(n = 2425)

Records excluded (n = 2325)

Full-text articles assessed for eligibility (n =100)

Full-text articles excluded (n = 78)

Reasons for exclusion:

Biomarker not modelled (n= 21)

Costing study (n= 1)

Letter (n= 1)

Not in English (n= 1)

Not intervention of interest (n= 5)

Not study type (n= 12)

Poster (n= 1)

Pre-specified population (n= 11)

Not in full text (e.g. abstract) (n = 25)

Duplicate publication (n=1)

Studies included
(n = 22)
