# Supplementary material for: How are we evaluating the cost-effectiveness of companion biomarkers for targeted cancer therapies? A systematic review
Source: BMC Cancer. 2021 Sep 1;21:980. doi: 10.1186/s12885-021-08725-4 (PMC8408935; doi:10.1186/s12885-021-08725-4)
Supplement: Supplementary file 4 — Additional file 4. List of including the characteristics of companion biomarkers in the economic evaluations. [file 12885_2021_8725_MOESM4_ESM.docx]

**Additional file 4. List of including the characteristics of companion biomarkers in the economic evaluations**

| **Study** | **Q1** | **Q2** | **Q3** | **Q4** | **Q5** | **Q6** | **Q7.1** | **Q7.2** | **Q8** | **Q9** | **Q10** | **Q11** | **Q12** | **Score^*^** |
| --- | --- | --- | --- | --- | --- | --- | --- | --- | --- | --- | --- | --- | --- | --- |
| Aguiar 2017 | Yes | Yes | Yes | Yes | No | No | No | No | No | Yes | No | Yes | No | 6 |
| Bhadhuri 2019 | No | Yes | Yes | Yes | No | No | Yes | Yes | No | No | Yes | No | Yes | 7 |
| Chouaid 2017 | No | Yes | No | No | No | No | No | Yes | No | No | Yes | No | No | 3 |
| Curl 2014 | No | Yes | Yes | No | No | No | No | Yes | No | No | Yes | Yes | No | 5 |
| Dottino 2019 | Yes | Yes | Yes | Yes | No | No | Yes | Yes | No | Yes | Yes | No | Yes | 9 |
| Ewara 2014 | No | Yes | Yes | No | No | No | No | No | No | No | Yes | No | No | 3 |
| Genuino 2019 | No | Yes | Yes | No | No | No | No | No | No | No | Yes | No | Yes | 4 |
| Graham 2014 | No | Yes | No | No | No | No | No | Yes | No | No | Yes | Yes | Yes | 5 |
| Graham 2016 | No | Yes | No | No | No | No | No | Yes | No | No | Yes | Yes | No | 4 |
| Harty 2018 | Yes | Yes | Yes | Yes | No | No | No | Yes | No | Yes | Yes | Yes | No | 8 |
| Holleman 2020 | No | Yes | Yes | No | No | No | No | Yes | No | No | Yes | No | No | 4 |
| Huxley 2017;  Tikhonova 2018 | No | Yes | Yes | No | No | No | No | Yes | No | Yes | Yes | No | Yes | 6 |
| Janmaat 2016 | No | Yes | Yes | No | No | No | No | Yes | No | Yes | No | No | Yes | 5 |
| Lim 2016 | Yes | Yes | Yes | Yes | Yes | No | No | Yes | No | Yes | Yes | Yes | Yes | 10 |
| Lu 2016 | Yes | Yes | Yes | Yes | Yes | No | Yes | Yes | No | Yes | Yes | Yes | Yes | 11 |
| Lu 2018 | Yes | Yes | Yes | Yes | Yes | No | No | Yes | Yes | Yes | Yes | No | Yes | 10 |
| Morgan 2017 | No | Yes | Yes | No | No | No | No | Yes | No | No | Yes | Yes | No | 5 |
| Saito 2017 | Yes | Yes | Yes | Yes | No | No | No | Yes | Yes | No | Yes | Yes | Yes | 9 |
| Wen 2015 | Yes | Yes | No | Yes | No | No | No | Yes | No | No | No | Yes | No | 5 |
| Westwood 2014 | Yes | Yes | Yes | Yes | Yes | Yes | Yes | Yes | No | Yes | Yes | Yes | Yes | 12 |
| Wu 2017 | Yes | Yes | Yes | Yes | No | No | No | Yes | No | Yes | Yes | Yes | Yes | 9 |
| Zhou 2016 | Yes | Yes | No | No | No | No | No | Yes | No | No | Yes | No | No | 4 |

*Scored based on the number of ‘yes’.
