# Supplementary material for: How are we evaluating the cost-effectiveness of companion biomarkers for targeted cancer therapies? A systematic review
Source: BMC Cancer. 2021 Sep 1;21:980. doi: 10.1186/s12885-021-08725-4 (PMC8408935; doi:10.1186/s12885-021-08725-4)
Supplement: Supplementary file 5 — Additional file 5. Summary of comparative analysis structure employed in EEs of companion biomarker therapies. [file 12885_2021_8725_MOESM5_ESM.docx]

**Additional file 5. Summary of comparative analysis structure employed in EEs of companion biomarker therapies**

| **No.** | **Study** | **Structure of strategy comparisons** | **Baseline**  **analysis** | **Secondary analysis** |
| --- | --- | --- | --- | --- |
| 1 | Aguiar 2017 | Treat-all with guided therapy vs. treat-all with non-guided therapy respectively for unselected patients and biomarker-specified patients with PD-L1 expression. | Group 5 | n/a |
| 2 | Bhadhuri 2019 | Treat-all with guided therapy vs. treat-all with non-guided therapy for biomarker-specified patients expressing high levels of PD-L1.  Test-treat vs. treat-all with guided therapy. | Group 5 | Group 2 |
| 3 | Chouaid 2017 | Treat-all with guided therapy vs. treat-all with guided therapy for biomarker-specified patients | Group 4 | n/a |
| 4 | Curl 2014 | Treat-all with non-guided therapy vs. treat-all with guided therapy. | Group 5 | n/a |
| 5 | Dottino 2019 | Test-treat with guided therapy vs. no treatment (observation – control strategy).  Test-treat with guided therapy vs. treat-all with guided therapy | Group 3 | Group 2 |
| 6 | Ewara 2014 | Treat all with guided therapy vs. treat all with guided therapy for biomarker-specified patients. | Group 4 | n/a |
| 7 | Genuino 2019 | Treat all with guided therapy vs. treat all with non-guided therapy for biomarker-specified patients. | Group 5 | n/a |
| 8 | Graham 2014 | Treat all with guided therapy vs treat all with guided therapy. | Group 4 | n/a |
| 9 | Graham 2016 | Treat all with guided therapy vs treat all with guided therapy | Group 4 | n/a |
| 10 | Harty 2018 | Treat all with guided therapy vs treat all with non-guided therapy for biomarker-specified patients.  *This HE study performed using individual patient data from CRYSTAL trial and this trial is for patients expressing the epidermal growth factor (EGF) receptor. [https://clinicaltrials.gov/ct2/show/NCT00154102] | Group 5 | n/a |
| 11 | Holleman 2020 | Treat all with guided therapy vs treat all with guided therapy for biomarker-specified patients. | Group 4. | n/a |
| 12 | Huxley 2017 | Treat all with guided therapy vs. treat all with non-guided therapy for biomarker-specified patients.  Secondary analysis – Cmab plus chemotherapy vs. Bmab plus chemotherapy. | Group 5. | Group 4 |
| 13 | Janmaat 2016 | Treat all with guided therapy vs. treat all with non-guided therapy. | Group 5. | n/a |
| 14 | Lim 2016 | Test-treat with guided therapy vs. treat all with non-guided therapy. | Group 3. | n/a |
| 15 | Lu 2016 | Test-treat with guided therapy vs. treat all with non-guided therapy. | Group 3. | n/a |
| 16 | Lu 2018 | Test-treat with guided therapy vs. treat all with non-guided therapy. | Group 3. | n/a |
| 17 | Morgan 2017 | Treat all with guided therapy vs. treat all with non-guided therapy for biomarker-specified patients with ALK-positive NSCLC. | Group 5. | n/a |
| 18 | Saito 2017 | Test-treat with comprehensive profiling vs. test-treat with RAS screening.  Secondary: test-treat with RAS testing and anti-EGFR therapy vs. treat all with anti-EGFR therapy and no testing. | Group 1. | Group 2. |
| 19 | Wen 2015 | Biomarker-specified group (FIRE-3 trial patients to be KRAS wild type) treated with the guided therapy vs. another guided therapy. | Group 4. | n/a |
| 20 | Westwood 2014 | Test-treat vs. test-treat | Group 1. | n/a |
| 21 | Wu 2017 | Test-treat with Cetuximab vs. treat all with chemotherapy without testing | Group 3. | n/a |
| 22 | Zhou 2016 | Biomarker-specified group (CALGB 80405 trial with KRAS wild type) treated with the guided therapy vs. another guided therapy. | Group 4. | n/a |
